# Nonviral Gene Delivery of Growth and Differentiation Factor 5 to Human Mesenchymal Stem Cells Injected into a 3D Bovine Intervertebral Disc Organ Culture System

**DOI:** 10.1155/2013/326828

**Published:** 2013-12-23

**Authors:** Christian Bucher, Amiq Gazdhar, Lorin M. Benneker, Thomas Geiser, Benjamin Gantenbein-Ritter

**Affiliations:** ^1^Institute for Surgical Technology and Biomechanics, Tissue & Organ Mechanobiology, Medical Faculty, University of Bern, Stauffacherstraße 78, 3014 Bern, Switzerland; ^2^Department of Pulmonary Medicine, University Hospital Bern and Department for Clinical Research, University of Bern, 3010 Bern, Switzerland; ^3^Department of Orthopaedic Surgery, Insel Hospital, University of Bern, 3014 Bern, Switzerland

## Abstract

Intervertebral disc (IVD) cell therapy with unconditioned 2D expanded mesenchymal stem cells (MSC) is a promising concept yet challenging to realize. Differentiation of MSCs by nonviral gene delivery of growth and differentiation factor 5 (GDF5) by electroporation mediated gene transfer could be an excellent source for cell transplantation. Human MSCs were harvested from bone marrow aspirate and GDF5 gene transfer was achieved by *in vitro* electroporation. Transfected cells were cultured as monolayers and as 3D cultures in 1.2% alginate bead culture. MSC expressed GDF5 efficiently for up to 21 days. The combination of GDF5 gene transfer and 3D culture in alginate showed an upregulation of aggrecan and SOX9, two markers for chondrogenesis, and KRT19 as a marker for discogenesis compared to untransfected cells. The cells encapsulated in alginate produced more proteoglycans expressed in GAG/DNA ratio. Furthermore, GDF5 transfected MCS injected into an IVD papain degeneration organ culture model showed a partial recovery of the GAG/DNA ratio after 7 days. In this study we demonstrate the potential of GDF5 transfected MSC as a promising approach for clinical translation for disc regeneration.

## 1. Introduction

Nonviral gene delivery is of great interest to stimulate cells for a direct potential clinical application for a wide range of musculoskeletal diseases. For repair and regeneration of the spinal intervertebral disc, cells would be required to match the native population in the intervertebral disc (IVD) niche. The nucleus pulposus cells, at the centre of the disc, and the annulus fibrosus cells, which populate the IVD “niches,” are difficult to reproduce in the laboratory since the unique markers to identify these cells are not known yet [1–4]. The IVD niche is defined as a low pH, very dense extracellular matrix consisting of collagen and glycosaminoglycan such as aggrecan and relative low cellularity [5, 6]; therefore the disc environment causes a major challenge for implanted cells such as MSCs. One strategy if cells are to be transplanted into a complex niche such as the IVD, which is populated by highly specialised and perfectly adapted native cell population, is to precondition the cells with some specialised growth factor (GF) cocktail (which is not known yet) or by additional mechanical stimuli [7]. Without doubt bone marrow derived mesenchymal stem cells (MSCs) have been proposed in many fields of musculoskeletal research because they can be isolated relatively easily, show a fast proliferation, and hold the potency to differentiate into different mesenchyme tissues [8, 9]. However, their usage for clinical applications has yet to be determined as there are safety restrictions and quality checks required before reimplantation into a patient [10, 11].

The current surgical approach is either to remove the disc (discectomy) and to fuse the adjacent vertebral bodies or to add a metal cage in place [12]. Biological therapies to repair the intervertebral disc are highly warranted. Growth factor treatment is assumed to be a promising approach for IVD regeneration. However, delivery to the target cells seems to be hindered and mainly unsolved as it has been reported for injection studies of BMP-2 in clinical study [13]. On the other hand, approach to use hydrogel for regenerating the centre of the disc has been proposed and studied numerous times with moderate outcome, especially in biomechanical properties to withstand mechanical loading in the long term [14]. Among the methods tried for gene transfer so far adenoviral methods [15, 16] have been widely used, which have high efficiency but are not very much accepted in the translational research perspective [17]. Growth and differentiation factor 5 (GDF5, syn. BMP14 or CDMP1) has been shown to be a key factor to push primary hMSCs towards an intervertebral-disc-like phenotype [18, 19], but it was also confirmed as a stimulating and a strong regenerative factor for disc cells *in vitro* [20] and *in vivo* in a rabbit IVD annulus stab degeneration model [21]. Further evidence for key importance of GDF5 for low back pain comes from a population based study to identify linkage disequilibrium of a single-nucleotide polymorphism [22]. Recombinant human rhGDF5 is currently under investigation as a drug to regenerate the IVD in a randomised phase-II clinical trial (http://www.clinicaltrials.gov/ case NCT01124006). However, exogenous injection of GDF5 is a very costly treatment with not a very endurable scenario unless it is delivered with a technique, which can offer sustained levels to achieve therapeutic benefits. The question of drug delivery to cells is unsolved as recently suggested by Carragee et al. for the case of direct injection of BMP2 as a spinal fusion enhancer [13]. Thus, we were interested to test nonviral gene therapy approach by direct delivery of the gene via electroporation of primary hMSCs and to test whether these would differentiate into intervertebral-disc-like precursor cells by overexpression of GDF5. Thus, we performed *in vitro* electroporation mediated gene transfer of GDF5 to primary hMSCs and injected them into an intervertebral disc explant model, with an aim to achieve disc regeneration.

## 2. Materials and Methods

### 2.1. Cell Source and Expansion

Human bone marrow was obtained from 12 patients aged 20–60 undergoing hip or spine surgery after written consent (Table 1). The procedure was approved by the Ethics Office of the Canton of Bern (KEK # 187/10). Human mesenchymal stem cells (hMSCs) were amplified from the mononuclear cell fraction after density gradient centrifugation (Histopaque-1077, Sigma-Aldrich, Buchs, Switzerland) by selection for plastic adherence for 1-2 passages. The hMSCs were expanded using Dulbecco's Modified Eagle Medium (DMEM), low glucose, GlutaMAX, and pyruvate with 10% FBS, 100 *μ*g/mL penicillin, 100 UI/mL streptomycin, and 2.5 ng/mL bFGF-2 [23].

### 2.2. Characterization of hMSCs for Presence of Stromal CD Marker Expression

The isolated human mesenchymal stem cells were characterised for their surface markers. Two independent MSC populations (2 different donors, patient 59 and 60) were randomly selected and analysed for CD105, CD44, CD29, CD90, and CD45 (Abcam, Cambridge, MA, USA) by flow cytometry on a FACS LSRII (BD Biosciences Inc., Brussels, Belgium). The results for each CD marker were compared to unstained cells and isotype controls.

### 2.3. Nucleofection of Cells

**Figure 1:**
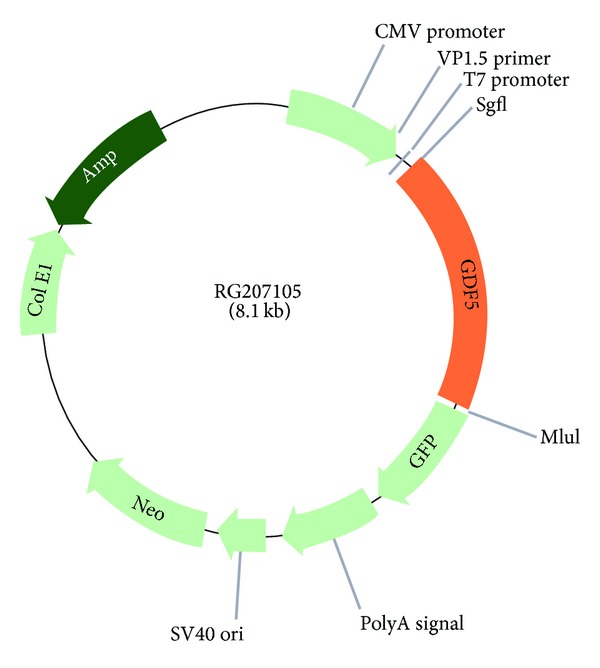
Plasmid map of pCMV6-AC-GFP vector containing the true gold ORF of human GDF5 (GenBank accession number NM_000557) with C-terminal TurboGFP.

The transfection-ready plasmid RG207105 Human cDNA ORF Clone of GDF5 (NM_000557) was selected as a vector. The plasmid contains a CMV promoter and a fusion protein of GDF5 including a GFP-tag (OriGene Technologies Inc., Rockville, MD, US) (Figure 1). Prior to electroporation RG207105 was amplified in *E. coli* with positive selection in presence of ampicillin (Sigma-Aldrich, St. Louis, USA) and purified using QuickLyse Miniprep Kit according to the instructions of the manufacturer (Qiagen Inc., Basel, Switzerland). The ORF of the sequence of GDF5 was back-checked for accumulation of point mutations resulting from cloning using direct DNA sequencing of the insert of RG207105 (Microsynth, Balgach, Switzerland) using the provided DNA primers. hMSCs were electrophoresed by Nucleofector technology (Amaxa, Lonza, Basel, Switzerland) using Lonza's optimized protocol U-23 (the protocol C-17 revealed low transfection efficiency of ~10% transfected cells) and the mesenchymal stem cell Nucleofector solution (VPE-1001, Amaxa, Lonza Inc.), which provided a protective environment and allowed high transfection efficiency and cell viability. About 500k cells were transfected per reaction, centrifuged, resuspended in Nucleofector solution, and mixed with 2 *μ*g of plasmid DNA according to the instructions of the manufacturers. Directly after the electrical pulses, prewarmed DMEM LG, 10% FBS was added to the cuvette and the cell suspension was seeded into a 6-well or 12-well plate.

### 2.4. Immunohistological Staining

Cells growing in monolayer were stained for the presence of intracellular GDF5. Transfected cells were seeded onto glass cover slips in a 6-well plate and cultured for 14 days. Then the cells were initially fixed in 4% paraformaldehyde (PFA) directly on the 6-well plate. Cells were then made permeable by incubation with 0.1% Tween in PBS. Cells were preincubated for 1 h with 1xPBS and 10% FCS to block the unspecific staining, washed, and then incubated with the primary rabbit polyclonal antibody (GeneTex, Ivine, CA, US, cat. Number GTX113580) against GDF5 at a dilution of 1 : 200 for 1 h. After thoroughly washing the cells the secondary red fluorescently labelled antibody (Abcam, ab6939) was amended at a dilution of 1 : 1000. The stained cells on the glass cover slip were mounted on a glass slide with 1 droplet of mounting medium for fluorescence with DAPI (Vectashield, H-1200, Reactolab, Servion, Switzerland). Images were taken with a fluorescence microscope AF 6000 LX from Leica (Wetzlar, Germany).

### 2.5. 3D Alginate Culture

Electroporated MSCs were trypsinized after 1 week of monolayer culture (expansion) using 0.5% EDTA-Trypsin (Gibco, Life Technologies, Basel, Switzerland). Cells were washed in phosphate buffered saline (PBS) and seeded in 1.2% alginate with a density of 2 × 10^6^ cells/mL and pressed through a syringe with 22 G needle. This procedure allowed to create ~30 *μ*L beads which were formed by dropping the alginate into a 102 mM CaCl_2_ solution. Cells were then grown in high glucose DMEM GlutaMAX containing pyruvate, 10% FBS, 1% penicillin/streptomycin (Gibco, Life Technologies, Invitrogen, Basel, Switzerland) in the absence of dexamethasone. Beads were snap-frozen on days 7, 14, and 21 and were analysed for gene expression and GAG/DNA content.

### 2.6. Relative Gene Expression Analyses

For gene expression, cells were either directly lysed or alginate beads were flash-frozen in liquid N_2_. Beads were then shattered into powder under liquid N_2_ and total RNA was extracted from the constructs with TRI reagent (Molecular Research Center) using a modified TRIspin method [24]. Total RNA (100–200 ng) was used for reverse transcription (RT) and subsequent PCR (RT-PCR) using gene-specific primers and probes [25, 26]. Reverse transcription was performed with Bio-Rad reverse transcription reagents (Bio-Rad, Glattbach, Switzerland). Real-time PCR was performed on an IQ-5 RT-PCR system (Bio-Rad, Reinach, Switzerland) using SYBR Green technology. Oligonucleotide primers (all from Microsynth, Balgach, Switzerland) have been designed with Primer Beacon Designer Software (Premier Biosoft Inc., Palo Alto, CA, USA) using nucleotide sequences taken from the GenBank database (Table 2). RT-PCR was quantified using the IQ-5 cycler software (Bio-Rad, Basel, Switzerland). A threshold value of fluorescence was set in the exponential phase of the amplification, and the number of PCR cycles needed for each sample to reach that level was recorded as the C_*t*_ value [27]. Gene expression was quantified by ΔC_*t*_ values using the relative quantification method (Applied Biosystems: User Bulletin number 2 ABI Prism 7700 Sequence Detection System, 2001), which normalises C_*t*_ values relative to the gene expression of a house-keeping gene (e.g., ribosomal 18S). ΔΔC_*t*_ values at day seven will then be estimated relative to day 0 and transformed into relative mRNA values using the formula 2-ΔΔC_*t*_ [28]. We screened the relative gene expression of major anabolic genes: aggrecan, collagen I, collagen II; and other recently published IVD marker genes [2, 18, 29] (Table 2).

### 2.7. GAG/DNA Ratio

Three beads or the IVD tissues were digested overnight at 60°C with 125 *μ*g/mL papain from papaya latex (P-3125, Sigma-Aldrich) in 5 mM Cysteine-HCl (30119, Fluka, Buchs, Switzerland), 55 mM Na-Citrate (71406, Fluka), 150 mM NaCl (71380, Fluka), and 5 mM EDTA (03685, Fluka). Glycosaminoglycan (GAG) content was quantified by 1,9-dimethylmethylene blue (DMMB) binding assay. DMMB binds to GAG at a pH of 1.5 (but alginate is believed to be protonated at pH 1.5) and absorption can be measured at a wavelength of 600 nm. Standard curve is done with a dilution of chondroitin sulfate (C9819, Sigma-Aldrich) in papain buffer, described above. The amount of DNA was determined by Quant-iT PicoGreen dsDNA Reagent (P11496, Invitrogen, Life Technologies Corp., Basel, Switzerland), following the manufacturer's protocol with a high-range standard curve of Lambda DNA standard (P11496, Invitrogen Life Technologies Corp., Basel, Switzerland) [30, 31]. Fluorescence was measured using a spectrofluorometer reader (SpectraMax, M5, Molecular Devices, USA) at excitation 487 nm and emission at 525 nm, with a cutoff at 515 nm.

### 2.8. *In Vitro* Organ Culture Model and hMSC Injection

In order to test the feasibility of cell survival in the IVD environment the transfected cells were injected into an established organ culture model. Bovine coccygeal intervertebral discs were harvested from a local abattoir according to regulations of the local authorities. Intervertebral discs were prepared for organ culture as described previously using Ringer solution and a jet lavage spraying technique to enable nutrient diffusion for organ culture [32, 33]. The prepared discs were then injected with 60 i.U. of papain (Sigma, Aldrich) using a 25 G needle in the center of the IVD as described previously and incubated for 7 days at 37°C in a standard incubator and HG-DMEM and 5% FCS in free-swelling conditions. The cells were taken up at a density of 4 M cells/mL in soft Q-Gel polyethylene glycol (PEG) hydrogel (Ref 1004, MMP-degradable matrix without RGD, Q-Gel SA, Lausanne, Switzerland). After the cavity has been formed with papain digestion about 200 *μ*L, thus about 200 k hMSCs were injected into the cavity using a 22 G needle and a 1 mL syringe (BD Bioscience, Allschwil, Switzerland). There were three groups of cells injected: (1) untransfected MSCs, (2) transfected with a control plasmid (pmaxGFP, Amaxa, Lonza, Basel, Switzerland), and (3) MSC+pRG207105 containing the GDF5 sequence. Additionally, there was a hydrogel only control and an untreated organ culture control disc. The IVDs with the injected hydrogel/cell mixture were then incubated for 7 days without loading in special designed culture chambers [33]. After incubation the IVDs were dissected and the hydrogel/cell mixture was inspected macroscopically and microscopically. The inner and outer annulus fibrosus of the IVD was subjected to analysis of GAG and DNA.

### 2.9. Statistics

The relative gene expression data were tested against a hypothetical mean of 1.0, which were the untransfected sham cells using a nonparametric Wilcoxon signed-rank test due to the nonnormal distribution of the data. All calculations were done in Prism 6.0 c (Palo Alto, CA, USA). For the GAG/DNA ratio we tested with an unpaired *t*-test assuming equal standard deviations and a one-sided tail.

## 3. Results

### 3.1. CD Marker Expression of hMSCs

Both selected donors showed positive staining for the markers CD90, CD44 and for one donor CD105 was also slightly positive. Negative staining was seen for both donors in the CD markers 45 (see Supplementary Figure 1 available online at http://dx.doi.org/10.1155/2013/326828).

### 3.2. *In Vitro* Transfection Assays in 2D and 3D

**Figure 2:**
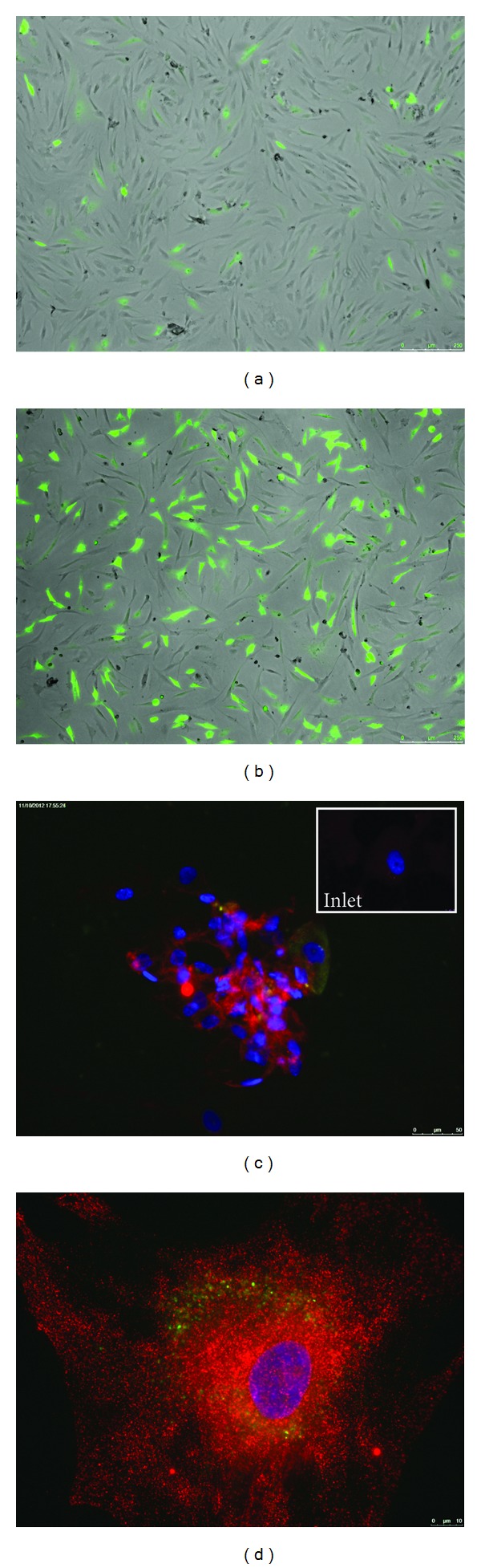
(a) and (b) pmaxGFP-transfected hMSC 48 hours after C-17 (a) or U-23 (b) electroporation using the Amaxa nucleofector. (c) and (d) GDF5 expression of transfected and control hMSC after 14 days of monolayer culture, anti-GDF5 antibody from GeneTex. Blue: cell nucleus stained by DAPI, green: translated GFP, red: intracellular GDF5. (c) Cell population at a resolution of 20x or (d) close-up (68x) hMSC transfected with RG207105, inlet in C = sham control.

**Figure 3:**
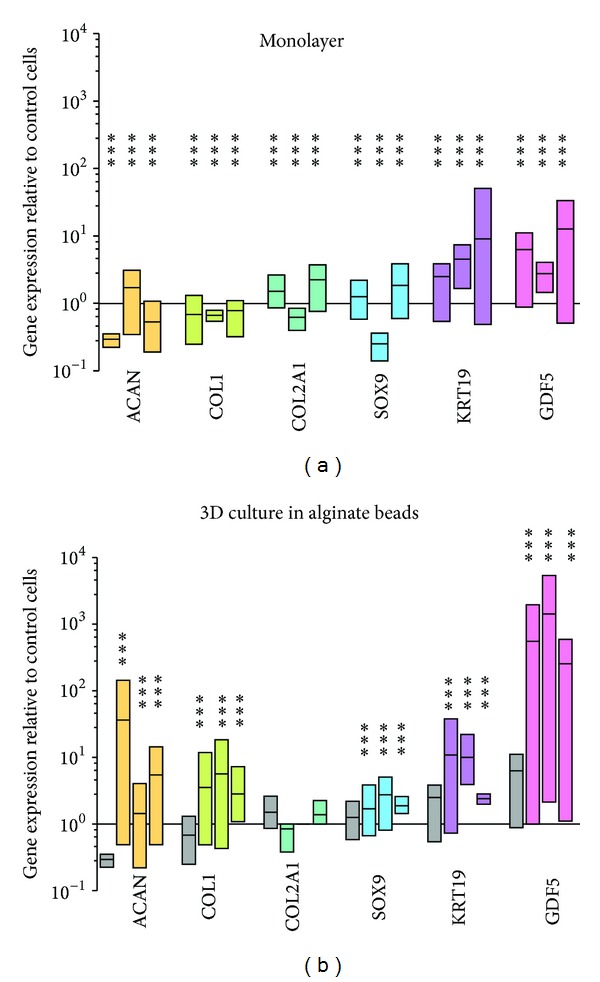
Relative gene expression to untransfected cells after 7, 14, and 21 days (three time points) in monolayer culture (a) and (b) in 3D cell culture using 1.2% alginate beads; gray bars correspond to state of day 0 cells (day 7 in monolayer culture). Bars represent min. to max. with a line at the mean. N = 4 ± SEM. *** denotes significantly different from 1 (untransfected cells) with *P* < 0.0001.

**Figure 4:**
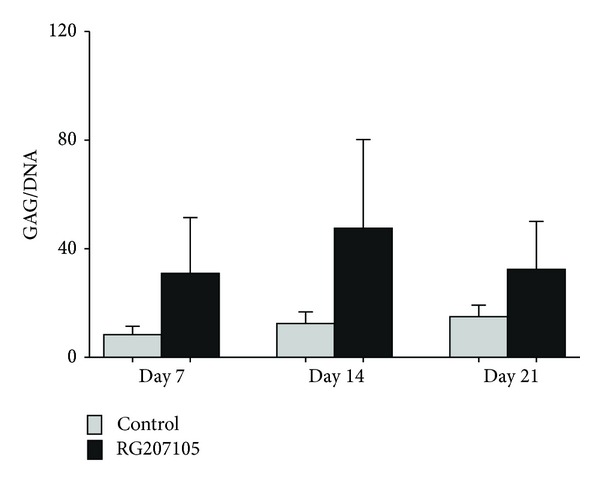
Glycosaminoglycan (GAG)/DNA ratio of alginate bead culture comparing transfected and untransfected cells over time.

MSC expressed GDF5 efficiently for up to 3 weeks. The fused protein could be found as a green fluorescence (GFP-tag), monitored under a green fluorescent microscope up to three weeks after transfection and could also be detected with the specific antibody against GDF5 (Figures 2(c) and 2(d)). The protocol U-23 produced significantly more green cells than C-17 (Figures 2(a) and 2(b)). The combination of GDF5 gene transfer and 3D culture in alginate showed a significant upregulation of SOX9 as a marker for chondrogenesis and KRT19 as a marker for discogenesis compared to untransfected cells (Figure 3). The hMSCs which were electroporated and then encapsulated in alginate and grown for 14 days tended to produce more proteoglycans in culture (GAG per DNA); however, this was not statistically significant, *P* = 0.196 (Figure 4).

### 3.3. 3D Organ Culture

**Figure 5:**
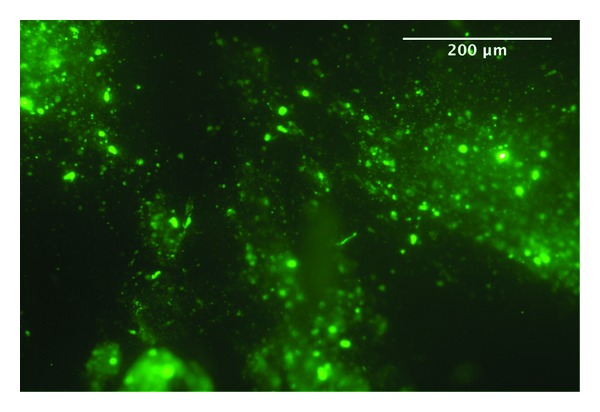
Q-Gel hydrogel with GFP expressing hMSCs (pmaxGFP) imaged with fluorescent microscope recovered from the center of the disc after 7 days of bovine intervertebral disc organ culture.

**Figure 6:**
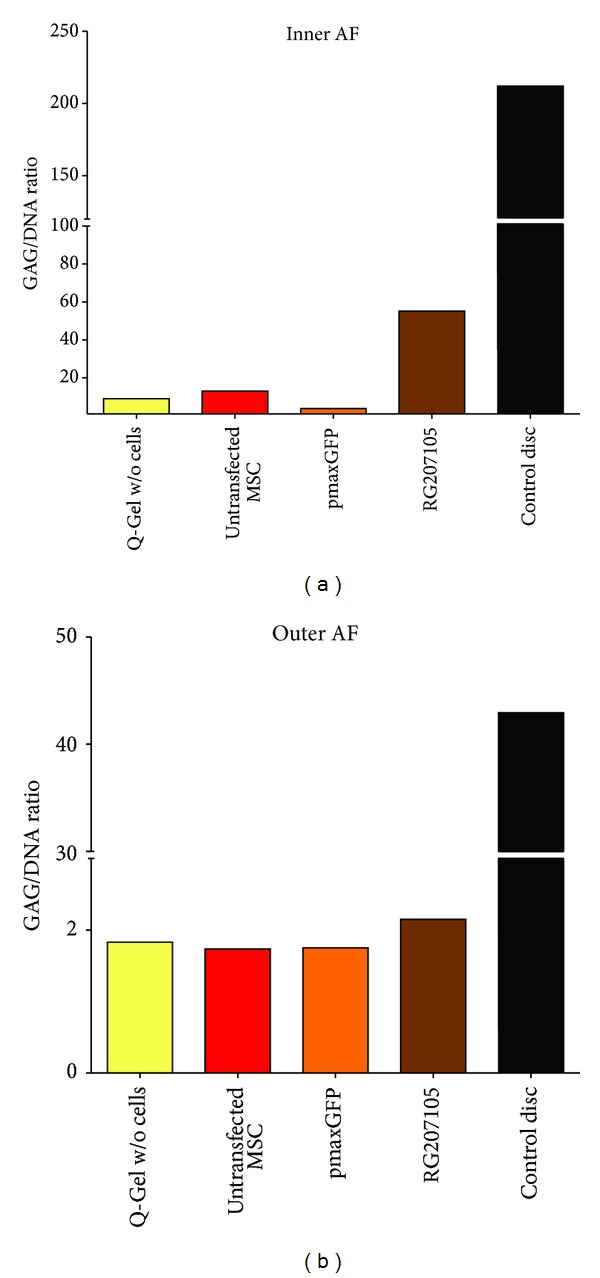
Glycosaminoglycan (GAG)/DNA ratio of outer and inner annulus fibrosus (AF) after 7 days of free-swelling organ culture of bovine intervertebral discs. Please note the difference between untransfected hMSC, control, pmaxGFP, and RG207105 transfected cells. The GDF5 overexpressing cells are pushing partially GAG/DNA ratio back in bovine IVD organ culture.

The incubation of cells and hydrogel inside the 3D organ culture revealed by macroscopic inspection that the PEG hydrogel shrunk considerably in volume to around 20% of its initial volume. However, the cells could be still detected in the hydrogel as imaged with fluorescent microscopy of the pmaxGFP transfected cells (Figure 5). The GAG/DNA ratio of papain digested discs was reduced considerably by almost a factor of 10 (Figure 6). However, in the group with the hMSCs, which were transfected with the RG207105 plasmid, a partial recovery of the GAG/DNA ratio could be observed (Figure 6).

## 4. Discussion

### 4.1. Phenotype of hMSCs

Our GDF5 transfected MCS differentiated towards an IVD-like phenotype. We found a significant upregulation of ACAN, SOX-9, and interestingly also KRT19. KRT19 has been recently identified as a potential marker for a discogenic phenotype [18, 19], and this “marker behaviour” of KRT19 could be confirmed in our study. However, the fact that collagen type 2 did not show any increase this might be a typical response of the growth factor GDF5 in absence of dexamethasone. Previous studies [18, 19] used dexamethasone in combination with the growth factors GDF5 and TGF-*β*, and dexamethasone has been shown to have stimulatory effects on its own [34, 35] in absence of TGF-*β* or other BMP-related cytokines. Thus, the current lack of collagen type 2 expression could be explained due to lack of dexamethasone or due to differential expression of GFP-tagged fusion protein compared to GDF5 alone (without GFP). Previous studies have demonstrated stimulatory effect on *in vitro* intervertebral disc cells [20, 36]. A similar approach was also undertaken by Wang et al. [16] but using an adenovirus as a vector. We also tested the plasmid pZS2GDF5, which Wang et al. [16] used previously. The relative gene expression results (data not shown) were similar as with the GFP-tagged plasmid RG207105. Interestingly the production of GAG increased in 3D cultures compared to the monolayer culture. GDF5 is an interesting candidate for gene therapy for the intervertebral disc as it has been shown in a rabbit annulus-puncture degeneration model [21].

The hMSCs utilized in our study are predominately from women donors (Table 1) and it should be mentioned that the outcome of donor variation might be biased. It has been recently reported in primary intervertebral disc cells [37] that only male nucleus pulposus cells (NPC) responded to testosterone present in the culture media. However, in hMSCs Bertolo et al. [37] did not find any sex-dependent effects of the donor variation. Moreover, a sex-dependent polymorphism of estrogen receptor alpha PvuII restriction site has been recently associated with the higher basal osteoblast differentiation capacity of MSCs [38].

The outcome of the organ culture feasibility study revealed that the transfected cells could have some potential to recover the GAG/DNA ratio (Figure 6), especially in the inner AF, the region where a cavity was created in the disc degeneration model. It is yet unclear how the phenotype of the injected hMSCs is progressing in the tested PEG hydrogel and the disc environment. Due to problems to extract enough RNA for RT-PCR reaction and/or problems from the PEG hydrogel we were unable to demonstrate the actual phenotype of hMSCs after 3D organ “coculture” of the cells. However, we speculated that the phenotype might have been even improved since the GAG/DNA ratio increased quite dramatically from 9.0 to 55.2 (Figure 6).

### 4.2. The Action of GDF5 during Musculoskeletal Development

There have been a number of studies performed to study the function of GDF5 onto the development of the skeletal system.

In a wild type mouse the phenotype of brachypodism appears in the E12.5 state of embryonic development. At this point the first cartilage condensations are formed, which then are reduced in the brachypod (bp) mouse and also the cartilage differentiation is delayed. However overexpression of GDF5 in the mouse [39] and chicken [40] leads to an increased cartilage condensation secondary to thickening of the cartilage Anlagen. These studies showed that GDF5 regulates the initiating cartilage formation with induction of cell adhesion and condensation of mesenchymal cells followed by the differentiation towards chondrocytes. In the course of development GDF5 controls the proliferation of those chondrocytes in the perichondrium and therefore has an impact on the growth and shape of the developing bones. GDF5 is also expressed in the future articular zones, where it has a high influence on the development of the joints [41, 42].

How essential GDF5 is for the development of the extremities can easily be seen on patients with a loss of function in GDF5. A heterozygous loss of function mutation in GDF5 leads to a malformation of the hands, the brachydactyly type C [43]. Homozygous GDF5 loss of function mutation leads to a more complex alteration of the skeleton also known as the group of acromesomelic chondrodysplasia. They are clinically classified as Grebe type, Hunter-Thompson type, and the DuPan type. Patients with Grebe type show the most severe form of alteration of the skeleton with a high degree dwarfism, extremely short extremities and rudimentary finger Anlagen [44]. Patients with the Hunter-Thompson type feature a similar phenotype however with a less severe manifestation [45]. The DuPan type is termed the mildest type of the acromesomelic chondrodysplasia. It implies as a clinical feature a fibular hypoplasia, which leads to severe disability of normal walking [46].

## 5. Conclusions

The present study could reproduce partially the stimulating effects of overexpressing GDF5 of GAG/DNA ratio obtained in previous studies using an adenoviral delivery system for nucleus pulposus (NP) cells or NP explants [16, 20, 40]. Here, we further show that overexpression of GDF5 will switch on expression of ACAN but not necessarily collagen type 2 in the absence of dexamethasone in the medium. However, this specific gene expression profile, which is induced by the GF, makes GDF5 a possible target candidate for the production of therapeutic cells grown from expanded hMSCs and reimplanted into the degenerated disc of a low back pain patient. Upcoming studies will investigate efficiency of nonviral gene transfer of multiple genes (i.e., GDF5 in combination with TGF-*β*) and combined effect of gene therapy and environmental conditions such as soluble GFs or hypoxia.

## Figures and Tables

**Table 1:** List of donors used for hMSC expansion. All cells were obtained with ethical approval (KEK 187#10) from bone marrow aspirates of the vertebral body of patients undergoing spinal surgery.

ID	Site of aspiration	Gender	Age
Patient 49	—	Male	51
Patient 50	—	Female	77
Patient 51	Th11, Th12	Female	38
Patient 52	L1	Female	79
Patient 53	—	Female	86
Patient 54	Th11, L1	Female	73
Patient 55	Th10, Th12	Male	61
Patient 56	Th11, L1	Female	58
Patient 57	Iliac Crest	Female	81
Patient 58	Th5, Th6	Female	54
Patient 59	Th10, Th12	Female	80
Patient 60	Th10, L2, L3	Female	81

Th: thoracic vertebral body, L: lumbar vertebral body.

**Table 2:** Primer list used for RT-PCR.

Abbreviation	Gene name	Forward	Reverse
Hs_18S	Reference gene	CGA TGC GGC GGC GTT ATT C	TCT GTC AAT CCT GTC CGT GTC C
Hs_ACAN	Aggrecan	CAT CAC TGC AGC TGT CAC	AGC AGC ACT ACC TCC TTC
Hs_COL1	Collagen1 A2	GTG GCA GTG ATG GAA GTG	CAC CAG TAA GGC CGT TTG
Hs_COL2A1	Collagen 2 A1	AGCAAGAGCAAGGAGAAG	GGGAGCCAGATTGTCATC
Hs_SOX9	SRY hox gene 9	GAG ACT TCT GAA CGA GAG	GGC TGG TAC TTG TAA TCC
Hs_KRT19	Keratin 19	TGT GTC CTC GTC CTC CTC	GCG GAT CTT CAC CTC TAG C
Hs_GDF5	Growth and differentiation factor 5 (GDF5)	ATCAGCATCCTCTTCATTGACTCT	ACACGACTCCACGACCAT
Hs_GFP	Green fluorescent protein	ATGACCAACAAGATGAAGAG	AAGTGGTAGAAGCCGTAG

## Abbreviations

GF: Growth factor
GDF5: Growth and differentiation factor 5, synonyms (BMP-14, CDMP-1)
FBS: Fetal bovine serum
ACAN: Aggrecan
COL1: Collagen type 1
COL2: Collagen type 2
hMSC: Human mesenchymal stem cells
KRT19: Cytokeratin 19
IVD: Intervertebral disc
ORF: Open reading frame.
